# Pain Changes Induced by Acupuncture in Single Body Areas in Fibromyalgia Syndrome: Results from an Open-Label Pragmatic Study

**DOI:** 10.1155/2021/9991144

**Published:** 2021-09-28

**Authors:** Marco Di Carlo, Giacomo Beci, Fausto Salaffi

**Affiliations:** Rheumatology Clinic, Università Politecnica Delle Marche, Jesi, Ancona, Italy

## Abstract

To date, there is considerable evidence of the effectiveness of acupuncture in fibromyalgia syndrome (FM). However, it is not known in which body areas acupuncture is more effective. The objective of this study was to assess the improvements of pain induced by acupuncture in single body areas in patients with FM. In this open-label pragmatic study, FM patients in a state of high disease severity were consecutively enrolled and treated with a course of 8 weekly sessions of manual acupuncture. Patients were assessed with the Self-Administered Pain Scale (SAPS) of the Fibromyalgia Assessment Status at baseline and at the end of eight acupuncture sessions. Acupuncture sessions were all conducted with the same acupuncture formula (LV3, SP6, ST36, LI4, CV6, CV12, Ex-HN-3, and GV20) in each session and in each patient. Ninety-six FM patients completed the course of treatment. All the 16 body areas assessed by SAPS showed improvement in pain. A statistically significant improvement was achieved in 12 of the 16 body areas investigated, with the best results in abdomen and forearms (*p* = 0.001), while the worst results were registered for neck (*p* = 0.058), chest (*p* = 0.059), left buttock (*p* = 0.065), and right thigh (*p* = 0.052). The treatment has also shown significant effectiveness in improving fatigue and sleep quality (*p* < 0.0001). Acupuncture has a beneficial effect on pain in all body areas in FM patients with high disease severity, with the greatest effects in the abdominal region and in the forearms, allowing a personalization of the treatment.

## 1. Introduction

Acupuncture, a therapeutic technique that goes back thousands of years, is still an effective treatment strategy for chronic musculoskeletal pain [1]. In particular, in the field of fibromyalgia syndrome (FM), there are growing clinical experiences oriented towards its integration into multimodal treatment schemes [2]. Indeed, the demand for integrating alternative therapy modalities into complex treatment regimes is growing rapidly, even for inpatients [3].

At present, randomized controlled trials (RCTS) have demonstrated the effect of acupuncture in the treatment of FM [4–7]. The latest EUropean League Against Rheumatism (EULAR) guidelines for the management of FM have suggested a “weak for” recommendation regarding the use of acupuncture [8]. The main advantages of acupuncture are the excellent tolerability and the almost absence of adverse events when compared to the standard drug therapy of FM [4]. A recent meta-analysis of RCTs documented the benefits of acupuncture on pain and quality of life in FM, demonstrating the superiority of verum acupuncture over sham acupuncture, and concluding that it may be a recommended treatment in FM patients [9].

However, in evaluating the effectiveness of acupuncture in FM, the majority of studies have focused mainly on generic patient-reported outcomes (e.g., numerical rating scale [NRS] of pain) [4], or disease-specific instruments (e.g., Fibromyalgia Impact Questionnaire [FIQ]) [5, 10]. Other authors have also documented improvements in serum biomarkers [6, 11].

The clinical manifestations of FM are numerous: they are proteiform, it is often difficult to frame them, and the complexity of the clinical picture is also responsible for a significant diagnostic delay [12]. However, the key symptom that characterizes FM is the presence of chronic widespread pain (CWP). The concept that FM is a condition primarily characterised by chronic pain is emphasised in the classification proposed by the International Classification of Diseases (ICD). In the 11th version of the ICD, FM is grouped into the conditions of chronic primary pain [13]. CWP remains the main criterion in the diagnostic definition of FM, although there are differences in its definition and assessment depending on the set of diagnostic/classification criteria used. Based on the criteria of the American College of Rheumatology (ACR) of 1990, the CWP was essentially evaluated by counting tender points [14]. The CWP was thus “measured” by the count of 18 myofascial tender points. Tender point count remained valid for many years, to be abandoned mainly because of the difficulties of objective assessment by general practitioners and the arbitrary nature of the assessment. Since 2010, with the new ACR diagnostic criteria, CWP evaluation has undergone a complete revolution with the introduction of the widespread pain index (WPI), a score between 0 and 19 in which the patient independently reports the presence or absence of pain in specific body regions [15]. The WPI has undergone some minor changes until the full definition of 2016 [16].

Recently, the ACTTION-American Pain Society-Pain Taxonomy (AAPT) initiative proposed a revision of the ACR criteria, introducing the concept of multi-site pain (MSP), which defines FM if present in at least six of the nine possible body areas [17].

Beyond the diagnostic definition and classification purposes of this complex sensory phenomenon that is CWP, there are no studies that have investigated in detail the improvements that acupuncture treatment can produce in different areas of the body. It is recognized that painful symptoms are more prevalent in some body regions in FM patients and occur more in the neck, upper back, and lower back [18].

For proper clinical application, it is important to identify whether the beneficial effects of a general acupuncture formula on pain are greater in certain body regions than others.

Starting from these considerations, the primary aim of this study is to assess the response to a general scheme of manual acupuncture, in terms of pain changes in single body areas in subjects with FM. The secondary aims of the study are to estimate the changes of fatigue and quality of sleep at the end of the treatment.

## 2. Materials and Methods

### 2.1. Setting and Study Design

This open-label, pragmatic, non-control study involved adult FM patients diagnosed according to 2010 ACR criteria [14]. Patients were consecutively recruited from January 2018 to June 2019 at a tertiary reference centre for the diagnosis and treatment of FM.

Patients underwent a course of eight weekly acupuncture sessions according to a pre-established schedule. The outcomes studied were assessed one week after the end of the acupuncture treatment course compared to baseline, i.e., at the beginning of the treatment course.

Acupuncture was not performed blindly and only one intervention group was chosen, in the absence of control groups. The reasons for this decision are explained later in Discussion.

All patients voluntarily participated in the study and signed informed consent. The study was approved by the local ethics committee (Comitato Etico Unico Regionale, number 1970/AV2) and conducted in accordance with the 1964 Helsinki Declaration and subsequent amendments.

### 2.2. Inclusion and Exclusion Criteria

For the purposes of this study, patients had to be in a status of severe FM, defined by the concomitant presence of a revised FIQ (FIQR) ≥39 and a Patient Health Questionnaire 15 items (PHQ15) ≥5 (Table 1 provides a synthetic description of the clinimetric instruments) [19].

In addition to these clinimetric criteria, drug therapy-related inclusion criteria were added. In particular, at the time of enrolment, patients had to show unresponsiveness or intolerance to reference drug therapy at stable dosages for at least three months. A combination of pregabalin 300 mg/day and duloxetine 60 mg/day was considered as reference drug therapy. Patients on lower dosages of the respective drugs were also admitted, provided that intolerance to titration of the drugs up to the dosages mentioned was recorded.

During the period of acupuncture treatment, patients were required to keep the reference drug therapy unchanged, while analgesics were allowed on demand (paracetamol up to 3 g/day or tramadol up to 150 mg/day).

All patients who had already undergone acupuncture even for indications outside FM, all patients with concomitant chronic painful conditions which could hamper patient evaluation or which could confound FM symptomatology (chronic inflammatory joint diseases, vasculitis, inflammatory muscle diseases or metabolic myopaties, thyroid diseases or other poorly controlled endocrinopathies, inflammatory bowel diseases, coeliac disease, Alzheimer's disease and other dementias, Parkinson's disease, and opioid-induced hyperalgesia), and all patients with relevant internistic diseases (uncontrolled heart failure, severe chronic renal failure, liver failure, and active infections) or cancer (uncontrolled active neoplasms) were excluded.

FM is frequently associated with other conditions such as lumbar or cervical spondylosis, peripheral osteoarthritis, or radiculopathies. These conditions were not considered as exclusion criteria as long as the dominant symptomatology was related to CWP.

### 2.3. Acupuncture Treatment Modalities

The procedures of this study were outlined according to the STandards for Reporting Interventions in Clinical Trials of Acupuncture (STRICTA) checklist [20].

The manual acupuncture sessions were all conducted by a single physician (MDC), a rheumatologist with nine years' experience in acupuncture, with the certification to practice acupuncture in accordance with Italian law obtained in 2014 at the end of a four-year training period.

**Figure 1:**
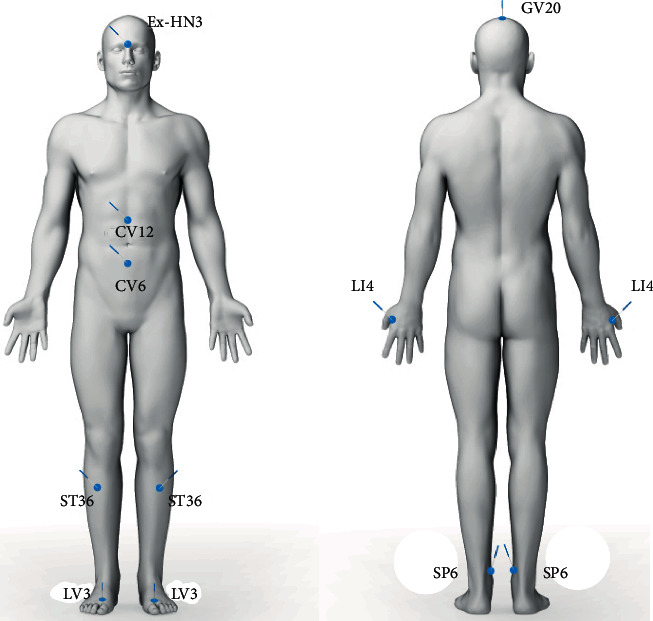
Front-back manikin with localization of acupoints.

The acupuncture scheme was performed according to the rules of Traditional Chinese Medicine (TCM). All patients were treated with the acupoints LV3, SP6, and ST36 on the lower limb, LI4 on the upper limb, CV6 and CV12 at abdominal level, Ex-HN-3 (Yintang), and GV20 at head level (Figure 1). Based on TCM, the proposed treatment formula aimed to move Qi, raise Qi, tonify Qi and blood, and calm Shen [21]. Pair acupoints were treated bilaterally. This acupuncture formula was performed in all patients for each treatment session, and all of the listed points were used simultaneously. Each point was inserted with sterile single-use needles 0.25 × 25 mm (Huanqiu®) equipped with a guide tube, and the needles were manipulated only at the beginning of the session until the so-called de Qi was reached. De Qi refers to those sensations (generally described as paresthetic sensations such as aching, soreness, numbness, tingling, fullness, distention, pressure, or heaviness) reported by the patient at the time of needle insertion and manipulation [22]. Each session lasted 30 minutes, and eight sessions were carried out weekly. In total, each patient received 240 minutes of acupuncture treatment.

The acupuncturist was allowed only minimal interaction with the patients at the beginning and end of the sessions, but was not allowed to make any questions about the state of health of the patients. The acupuncturist had no clinical and clinimetric information about the patients throughout the study.

### 2.4. Clinimetric Assessment

For the objectives of this study, the clinimetric evaluation was focused on Fibromyalgia Assessment Status (FAS) (Table 1) [23]. FAS has proven to be a valid, reliable, and responsive tool in the assessment of FM patients. Recently, a modified version of FAS has been used with excellent results also for diagnostic purposes [24].

FAS contains three evaluations: two 11-point NRS scales related to fatigue and sleep quality and the Self-Administered Pain Scale (SAPS). This latter scale was the one used for the primary aim of this study. SAPS evaluates 16 body regions for the presence of pain on 4-point NRS for each area, where 0 represents no pain, 1 mild pain, 2 moderate pain, and 3 severe pain. The reference to painful symptoms is relative to the last week. SAPS is administered on paper with a front and back representation of a manikin, and for the 16 body areas the patient indicates the pain level in the scales from 0 to 3 for each area. The final SAPS score ranges from 0 to 48 and is transformed by a nomogram to a 0–10 scale to be computed within the FAS. In this study, the improvement of pain symptoms in the single body areas was evaluated on the SAPS.

All questionnaires were administered by a fellow in rheumatology (GB), with experience in managing patient-reported outcome measures.

### 2.5. Statistical Analysis

For the purposes of this study, no formal sample size assessment was carried out. The case study enrolled is of convenience, determined by reasons of feasibility.

The data of this study showed a nonparametric distribution (verified with the Kolmogorov–Smirnov test); therefore, the results are presented as median and interquartile range for FAS and subscales. The homogeneity of the case study was assessed with Levene's test for SAPS, FAS fatigue, and FAS sleep quality.

In order to estimate the effect of acupuncture in each of the 16 body areas of the SAPS, and also to provide a graphical representation of acupuncture effects through a spidergram, mean SAPS values, not transformed by the nomogram, were calculated for each area at baseline and at the end of treatment.

Changes between baseline and endpoint were evaluated using the Wilcoxon test for paired data.

The statistical analysis was conducted with MedCalc, version 18.0.0, and statistical significance was considered for values of *p* < 0.05.

## 3. Results

The analysis was conducted on 96 of the 102 patients (94.1%) who started acupuncture treatment. In six patients, the course was suspended, at the second session in two patients due to poor tolerance to needle insertion, during the following sessions in the other four patients due to intervening logistics difficulties in carrying out the sessions on a weekly basis. Among the 96 patients treated, 85 (88.5%) were women and 11 (11.5%) men, with a mean age (±standard deviation) of 50.6 ± 12.3 years and a disease duration of 5.6 ± 6.2 years. The reference “optimal” drug therapy (combination of duloxetine and pregabalin) was taken by 32 (33.3%) patients, 22 (22.9%) patients were taking only pregabalin, 13 (13.5%) patients were taking only duloxetine, and 29 (30.2%) patients were not taking either molecule.

SAPS and FAS sleep disturbances data were homogeneous, whereas FAS fatigue data were not homogeneous.

SAPS, considered in its entirety, showed a significant improvement at the end of the acupuncture treatment, with median values that reduced from 5.8 at the beginning to 4.4 at the end of the course (*p* < 0.0001) (Table 2). Analyzing the data from the non-normalized and split SAPS in each body area, at baseline the highest mean scores were found at the axial skeleton level, with 2.24 (range 0–3) for the low back and 2.20 for the neck, respectively.

**Figure 2:**
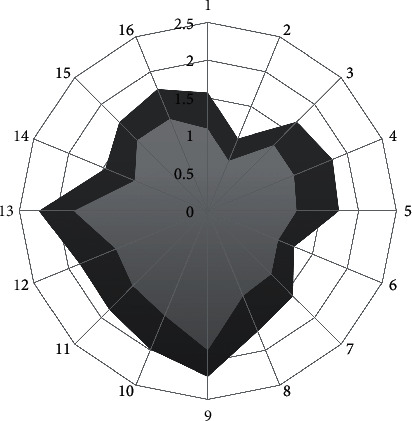
Spidergram showing improvement in final mean values (light grey spidergram area), after acupuncture treatment, compared to initial mean values (dark grey spidergram area) in the 16 body areas assessed by the Self-Assessment Pain Scale. 1 = head; 2 = chest; 3 = left arm; 4 = left forearm; 5 = abdomen; 6 = left buttock; 7 = left thigh; 8 = left leg; 9 = neck; 10 = upper back; 11 = right arm; 12 = right forearm; 13 = low back; 14 = right buttock; 15 = right thigh; 16 = right leg.

Comparing the mean values of the 16 body areas between basal and final, an improvement was recorded for each body area (Figure 2), with a mean difference of −0.43 points. A statistically significant improvement was achieved in 12 of the 16 body areas investigated, with the best results in abdomen and forearms (*p*=0.001). The four areas where the statistical significance of the improvement was not achieved were neck (*p*=0.058), chest (*p*=0.059), left buttock (*p*=0.065), and right thigh (*p*=0.052) (Table 3).

With regard to the secondary objectives of the study, fatigue and sleep quality showed also a significant improvement at the end of treatment compared to baseline (*p* < 0.0001) (Table 2).

## 4. Discussion

To the best of our knowledge, this is the first study that investigated the effects of acupuncture, in terms of pain improvement, in the single areas of the body in patients with FM. This study showed that improvements induced by a general acupuncture formula are widespread, even in areas of the body not directly stimulated by needles. However, in some areas improvements are greater. It can therefore be affirmed that acupuncture has a global effect on CWP, the key symptom experienced by FM patients. Acupuncture demonstrated also to be effective to treat fatigue and sleep disturbances.

Chronic musculoskeletal pain is the main reason why patients rely on acupuncture, followed by systemic symptoms such as fatigue [25, 26]. The effect of acupuncture in chronic pain is clinically relevant as demonstrated by the metanalysis of randomized controlled trials, and true acupuncture is more effective than sham acupuncture and non-acupuncture [27]. The mechanisms underlying the effectiveness of acupuncture in pain are multiple. Analgesia derives from integrated mechanisms involving the nervous system, the painful stimuli, and the stimuli resulting from skin infission with needles. Several neurotransmitters are involved, including serotonin, adenosine, gamma-aminobutyric acid, opioid peptides, calcitonin gene-related peptide, and substance P [28]. The above list of substances involved in acupuncture-induced analgesia is certainly not exhaustive, but the molecules act through pathways involving segmental and supraspinal spinal mechanisms [29].

These integrated mechanisms ensure that, through a general formula in accordance with the TCM, such as the one applied in this study, generalized benefits can be achieved. For example, improvements can also be documented in areas of the body where no needles are inserted, such as the lumbar region. This finding has important clinical consequences because, while it is essential that the treatment is effective, it must be optimally tolerated and, specifically, the formula used in this study allows the patient to lie comfortably on his back during the sessions.

However, it should be noted that the biggest improvements in terms of SAPS score difference between final and baseline were at abdominal level, where two acupoints (CV6 and CV12) were used. Our acupuncture formula included the treatment of 12 acupoints per session. Within the context of CWP of FM, where painful symptoms are also connoted by the presence of allodynia and hyperalgesia (as well as a major psychological burden), it is essential to balance the use of needles since it is virtually impossible to treat every single painful body area. Probably the integration of a general therapeutic scheme such as or similar to that used in this study, with a microsystem-based technique (e.g., auricular acupuncture, focusing on areas corresponding to the cervical region, where minor improvements were recorded), can provide even better results in terms of efficacy. However, this represents a theoretical speculation that should be the topic of future research.

The use of SAPS as a clinimetric instrument has allowed a detailed analysis of the changes induced by acupuncture in each body area. There are several ways to measure CWP, such as the WPI in the ACR 2016 criteria or the AAPT criteria manikin [16, 17]. In this study, we preferred to use the SAPS. This tool provides a synthetic result but also analyzes the pain in each body area, allowing the patient to describe it in 16 NRS. The result is a detailed mapping of the CWP. The studies that have verified the effectiveness of acupuncture on pain in FM are several; however, in our view, they have done so in an overly synthetic way, most of the cases using only a single VAS or NRS scale [4–6]. The description of a sensory phenomenon as complex and multifaceted as CWP in FM cannot be reduced to a single scale. Targino and colleagues revealed that the positive effects of acupuncture last for more than six months after the end of the treatment. In addition to general measures of quality of life, the beneficial effects of acupuncture showed also to reduce pain pressure threshold at tender points [7]. Mist and Jones documented in their RCT that acupuncture is superior to other non-pharmacological modalities such as patient education, demonstrating improvements in all generic and disease-specific indices [5]. Acupuncture demonstrated improving biochemical parameters, such as increased serotonin levels and reduced substance P [6, 11].

Not least, our study confirmed the effectiveness of acupuncture on fatigue and sleep quality, the other two variables considered in FAS. It should also be emphasised that this cohort of patients belonged to a category with severe symptoms refractory to conventional pharmacological therapy.

To date, there are no universally accepted treatment modalities for FM. What is widely accepted is the need for multimodal treatment approaches. Multimodal approaches, also suggested by international recommendations, are particularly useful for personalization of treatment, which is so important in FM, especially in patients with long-lasting symptoms [8, 30].

This study confirms that acupuncture, integrated with pharmacological therapy, is beneficial for pain management in FM patients. Several studies have already demonstrated the effectiveness of acupuncture in FM. However, no previous study had demonstrated improvement in pain in individual body areas.

Citing the limitations of the study, a first criticism that can be made is that no control group was used, in particular no group in which sham acupuncture was employed. We have chosen to treat all patients, first of all for the severity of their symptoms, since true acupuncture showed to be more effective than sham acupuncture in FM from recent meta-analyses [9, 31]. Secondly, based on a certain orientation in the literature, the effectiveness of acupuncture in chronic pain should be investigated with pragmatic real-life studies. Acupuncture represents a complex intervention that cannot be compared to a non-inert placebo such as sham acupuncture, which adds potential bias instead of removing it [32, 33]. A second criticism may be the single-centre recruitment. However, most of the studies dedicated to acupuncture are monocentric and this fact may also represent a strength since a certain uniformity of treatment has been guaranteed. A third criticism could be related to the acupuncture formula used, as one of several possible formulas was used. Virtually, the possibilities of combining acupoints are unlimited. We used a scheme in accordance with TCM, effective, with acupoints well known by acupuncturists, and easily repeatable in every patient.

A fourth limitation may be the fact that most of the patients were taking pharmacological treatment. The integration of acupuncture into multimodal schemes makes it more difficult to assess its therapeutic effect. However, all patients came from a failure/intolerance of drug therapy.

## 5. Conclusion

This study showed how eight sessions of manual acupuncture treatment, using a general formula in accordance with TCM, has whole body beneficial effects on CWP in FM patients. Improvements can be documented in each body area, with the greatest effects in the abdominal region and in forearms. Acupuncture is confirmed as a complementary method that can be effectively integrated with other treatment strategies in FM.

## Figures and Tables

**Table 1:** Description of the clinimetric characteristics of the instruments used as inclusion criteria (FIQR and PHQ15) and as outcome assessment (FAS).

Instrument	Number of items	Calculation	Interpretation
FIQR	21 items investigating 3 domains of health: 9 physical function, 2 overall health status, 10 symptoms	Each item is an 11-point NRS. The final score is the algebraic sum of the scores of the three domains: the sum of the 9 NRS in the physical function domain is divided by 3, the 2 NRS in the overall health status domain are considered as they are, and the sum of the 10 NRS of the symptoms domain is divided by 2.	Final score ranging from 0 to 100. Disease severity is defined as follows: remission (below 23), mild disease (between 24 and 40), moderate disease (between 41 and 63), severe disease (between 64 and 82), and very severe disease (above 83).

PHQ15	15 items, 13 investigating somatic symptoms, 2 psychological symptoms	Each item is scored from 0 (no disturbance) to 2 (severe disturbance). The final score is the algebraic sum of the items.	Final score from 0 to 30. The severity of symptoms is considered as follows: low (below 5), medium (between 5 and 10), and high (above 15).

FAS	It contains 2 parts. The first is made by 2 11-point NRS investigating fatigue and sleep quality, and the second is the SAPS. SAPS evaluates 16 body regions for the presence of pain on 4-point NRS (0 = no pain, 1 = mild pain, 2 = moderate pain, 3 = severe pain) for each area.	The final score is the mean of the algebraic sum of the 2 NRS for fatigue and sleep quality and the SAPS normalized (SAPS is scored from 0 to 48; then score is normalized to a 0–10 scale).	Final score from 0 to 10. No cut-off points for disease severity states are available.

FIQR = revised Fibromyalgia Impact Questionnaire; PHQ15 = Patient Health Questionnaire 15 items; FAS = Fibromyalgia Assessment Status; SAPS = Self-Assessment Pain Scale, NRS = numerical rating scale.

**Table 2:** Data at baseline and at the end of the acupuncture treatment regarding FAS and its subscales.

	Baseline		End of acupuncture treatment		Significance (*p*)^*∗*^
	Median	Interquartile range	Median	Interquartile range	
FAS total score	7.45	5.35–8.30	5.05	3.55–7.05	<0.0001
Fatigue	8.00	6.75–9.00	7.00	4.00–8.00	<0.0001
Sleep quality	8.00	4.75–9.00	6.00	3.00–8.00	<0.0001
SAPS	5.80	4.60–7.10	4.40	2.65–5.85	<0.0001

^*∗*^ = Wilcoxon test (paired samples). FAS = Fibromyalgia Assessment Status; SAPS = Self-Assessment Pain Scale.

**Table 3:** Mean values at baseline and at the end of acupuncture treatment for each of the 16 numerical rating scales (range 0–4, where 4 identifies the most severe painful symptoms) that describe body areas considered in the SAPS.

SAPS bodily areas	Mean values baseline	Mean values end treatment	Difference	Significance (*p*)^*∗*^
1. Head	1.57	1.09	−0.48	0.008
2. Chest	1.04	0.72	−0.32	0.059
3. Left arm	1.67	1.24	−0.43	0.002
4. Left forearm	1.79	1.24	−0.55	0.001
5. Abdomen	1.74	1.18	−0.56	0.001
6. Left buttock	1.24	1.01	−0.23	0.065
7. Left thigh	1.60	1.19	−0.41	0.009
8. Left leg	1.73	1.22	−0.51	0.005
9. Neck	2.20	1.85	−0.35	0.058
10. Upper back	1.99	1.50	−0.49	0.001
11. Right arm	1.85	1.41	−0.44	0.011
12. Right forearm	1.84	1.32	−0.52	0.001
13. Low back	2.24	1.78	−0.46	0.009
14. Right buttock	1.47	1.05	−0.42	0.012
15. Right thigh	1.66	1.33	−0.33	0.052
16. Right leg	1.75	1.32	−0.43	0.009
			Large sample test statistic *Z*, 3.558550	
			Two-tailed probability, *p*=0.0004^*∗∗*^	

^*∗*^ = Wilcoxon test (paired samples); ^*∗∗*^ = *p* value for nonnormalized SAPS. SAPS = Self-Assessment Pain Scale.

## Data Availability

The data are available upon reasonable request to the corresponding author.
